# Forkhead-box transcription factor 1 affects the apoptosis of natural regulatory T cells by controlling Aven expression

**DOI:** 10.1186/s12865-017-0198-8

**Published:** 2017-03-10

**Authors:** Zhitao Cai, Hong Liu, Xiongfei Wu

**Affiliations:** Department of Nephrology, Southwest Hospital, Third Military Medical University, Chongqing, 400038 People’s Republic of China

**Keywords:** Treg cells, Foxo1, Aven, Apoptosis

## Abstract

**Background:**

Regulatory T (Treg) cells play important roles in autoimmune diseases, cancer, and organ transplantation. Forkhead box protein o1 (Foxo1) and IL-7Rα(CD127) are closely related to the homeostasis of Treg cells. However, the mechanism underlying Treg proliferation and activation remains unclear. Here, we evaluated how the over-expression of Foxo1 affects Treg cell proliferation via intracellular signaling. nTreg cells were transfected separately with Foxo1 and Aven small-interfering RNA (siRNA) or over-expression plasmid. The expression of signaling pathway genes and CD127 was confirmed using RT-qPCR and western blot analysis. The expression of cell surface molecules and apoptosis was confirmed by Flow Cytometry 3-(4, 5-Dimethylthiazol-2-yl) 2,5- diphenyltetrazolium bromide for cell proliferation assays.

**Results:**

Foxo1 strengthened the proliferative ability of Treg cells by activating IL-7/CD127 signaling. In addition, Foxo1 suppressed Treg cell apoptosis by regulating Aven expression.

**Conclusions:**

The results in this study indicated that Foxo1 is a positive regulatory factor for the proliferation and activity of Treg cells. Foxo1 might be a potential target for the activation of nTreg cells in vivo and in vitro.

## Background

Regulatory T cells (Tregs) are a subpopulation of T cells that are thought to be derived from the same lineage as naïve CD4 cells due to the same expression of CD4 and CD25 [1]. In addition, Tregs express the biomarker forkhead box P3 (Foxp3) transcription factor [2, 3]. It has been reported that TGF-β is essential for Tregs to differentiate from naïve CD4 cells and is important in maintaining Treg homeostasis [4]. Tregs can be divided into two subsets: naturally occurring or thymus-derived Treg (nTreg) cells and T regulatory type 1(Tr1) cells. Tregs play an important role in autoimmune diseases, cancer, and organ transplantation [5–7].

Tregs are the front-runners in the race for therapeutic immune-regulation due to their ability to suppress effector T cells, which are known to play an important role in preventing autoimmunity [5, 8]. In recent years, it has been reported that nTregs can also effectively prevent the rejection of transplanted allografts in experimental models [9, 10]. Naturally, nTregs were used in hematopoietic stem cell transplantation to inhibit graft-versus-host disease (GVHD) [11, 12]. However, the widespread clinical use of Tregs has been limited by the low number of these cells in the periphery and immune homeostas [13]. Therefore, there is a great need for understanding the mechanism of proliferation and immune homeostasis in nTregs to prevent the rejection of transplanted allografts.

Interleukin-7 (IL-7) is a hematopoietic growth factor that plays a critical role in regulating the homeostasis of T cells [14, 15]. The control of IL-7 signaling is mainly dependent on the IL-7 receptor (IL-7R), which is a heterodimer that consists of the IL-7 receptor alpha (IL-7Rα, CD127) and common gamma chain receptor [16]. Therefore, CD127 plays a key role in modulating the homeostasis of T-cells. However, the molecular mechanism by which CD127 controls Treg cell proliferation and homeostasis remains unknown.

Forkhead box protein o1 (Foxo1) is a transcription factor and is characterized by a distinct fork head domain. It was shown that Foxo1-deficient mice developed a fatal inflammatory disorder [17]. Indeed, a growing body of research suggests that Foxo1 plays an important role in the immune system, including peripheral T-cell homeostasis [18]. Foxo1 can modulate the expression of IL-7Rα, which is expressed in T-cells [19]. Furthermore, Foxo1 can regulate the expression of Aven [17], which is an adaptor protein that has been implicated in anti-apoptotic signaling [20]. Therefore, we speculated that Foxo1 modulates Treg cell homeostasis and functions via CD127 and Aven.

In the present study, we found that Foxo1 over-expression resulted in Treg cell proliferation by activating IL-7/CD127 signaling. In addition, Foxo1 over-expression suppressed Treg cell apoptosis by regulating Aven expression. Taken together, these data suggest that Foxo1 is required for Treg cell proliferation and apoptosis by controlling CD127 and Aven expression.

## Methods

### Mice

Foxp3-GFP mice were obtained from Jackson Laboratories and were bred according to the Experimental Animal Centre of the Third Military Medical University and housed in a specific pathogen-free facility. Mice ages 6–8 weeks were used for the experiment. The experimental protocol was approved by the Ethics Committee of the Third Military Medical University.

### Sorting and flow cytometry

Spleen lymphocytes were isolated from Foxp3-GFP mice. The mice were killed by neck dislocation after following 4.5 mg/kg pentobarbital sodium (Sigma-Aldrich, St. Louis, MO, USA) via intraperitoneal injection in sterile environments. The spleen was removed for broking, and the cell lymphocyte suspension was obtained. Next, the cells were separated by lymphocyte separation liquid (TBD, Tianjin, China), and the cells were resuspended in FACS buffer (PBS) containing 1% BSA and 2% FBS for sorting by FACSCanto (BD Bioscience). The sorted cells were cultured in 1640 medium containing 20% FBS for further experiments.

Cell staining was performed in FACS buffer (PBS) containing 1% BSA and 2% FBS. The cells were stained with phycoerythrin-conjugated anti-mouse ICOS antibody (ebioscience, San Diego, CA, USA), Allophycocyanin conjugated anti-mouse CD127 antibody (BD Biosciences, San Jose, CA, USA), phycoerythrin-conjugated anti-mouse CD103 antibody (BD Biosciences) antibody or Allophycocyanin conjugated anti-mouse CD25 (Biolegend, San Diego, CA, USA) antibody at 4 °C for 30 min and were then washed with PBS containing 2% BSA or FBS (wt/vol). Cells were collected using a FACSCanto (BD Bioscience) and analyzed by FlowJo.

### Plasmid construction and transient transfections

Invitrogen provided Foxo1 and Aven small hairpin RNA (shRNA). With reference to the target gene, mouse Foxo1 Sense 5′ -GGG GTA TGG CCG AAG CGC CCC AGG -3′, antisense 5′- TTA GCC TGA CAC CCA GCT GAG AGC -3′, mouse Aven Sense5′ –GGG GTA TGT TCG AAG CAC GT -3′, antisense 5′-TCA GGA AAT CAT GCT GTA GAG CA-3′. A series of chemically synthesized oligonucleotides was spliced by polymerase chain reaction (PCR) to obtain gene sequences, and these sequences were then inserted into the pCMV5 vector [21]. The integrity of all constructs was confirmed by DNA sequencing. Using the Nucleofector Program U-25, 2 mg of plasmid was transfected into 1 × 10^6^ cells using the Amaxa Basic Nucleofector kit (Lonza, Switzerland), according to the manufacturer’s protocol.

### Quantitative PCR

Total RNA was extracted in Trizol LS reagent (Takara, Japan) and reverse-transcribed using a PrimeScript RT reagent kit (Takara). Next, cDNA was amplified (38 cycles of 95 °C for 20 s, 62 °C for 15 s, and 72 °C for 20 s) with SYBR qPCR SuperMix (Novoprotein, Shanghai, China) according to the manufacturer’s instructions, using gene-specific sets of primers: f for mouse Foxo1 gene, 5′ – TGT TTG ATT CAT TTC CTT TGG T -3′ and 5′ –TGA TTT TCT CCG CTT ACT GTT G -3′; mouse CD127 gene, 5′-AAA AGT AAA GCA TGA TGT GGC C -3′ and 5′ –TTG AAG TAA TCG TTA TGG GGA A -3′; mouse Icos gene, 5′ – CAT TCC CAA CAC GAA CAC CTA A -3′ and 5′ –TCT TCA CCC CCA GAA AAC ACA G -3′; mouse Aven gene, 5′- GGG ACC AGG AAC CAG AAA AAG A -3′ and 5′ – TAC ACA GAA GGC AAC CAG CAT T -3′; mouse IL-2 gene, 5′- GAT GAA CTT GGA CCT CTG CG -3′ and 5′- AGG GCT TGT TGA GAT GAT GC -3′; mouse IL-4 gene, 5′- CAT CCT GCT CTT CTT TCT CG -3′ and 5′- CCT TCT CCT GTG ACC TCG TT -3′; mouse IL-7 gene, 5′- GTT ATG GCA AAG CCA GAG CG -3′ and 5′- TGC GGG AGG TGG GTG TAG TC-3′; mouse IL-14 gene, 5′- CCC CTT CTG TCC AGC CAC TC -3′ and 5′- TCC CGT CTT CG TCC AA TCT-3′. mouse Bcl2 gene, 5′-GC TAC CGT CGT GAC TTC GC -3′ and 5′- ATC CCA GCC TCC GTT ATC C-3′. For each gene, the mRNA level was normalized against GAPDH expression in the respective cDNA preparation. Controls were set to 1.0, and each gene induction was calculated as the fold difference compared to controls. Each reported expression value represents the average of 3 independent experiments.

### Western blot analysis

Cells were collected and dissolved in RIPA buffer (1% sodium deoxycholate, 1% Triton X-100, 150 mM NaCl, 0.1% SDS, 10 mM Tris–HCl pH 7.2), Total proteins were separated on 10% SDS-PAGE and transferred onto polyvinylidene fluoride (PVDF) membranes by electroblotting for 1 h (110 V). The PVDF membranes (Millipore, MA, USA) were blocked in Tris- buffered saline containing 5% nonfat milk and incubated with primary antibody in Tris-buffered saline and Tween (TBST) with 5% nonfat milk over-night at 4 °C. Primary antibodies against the following proteins were used: Erk1/2, p-Erk1/2, Akt, p-Akt, Stat5, p-Stat5, Aven and GAPDH (Abcam, Cambridge, MA, USA); Foxo1, p-Foxo1 (Cell Signaling Technology, Danvers, MA,USA), CD127 (Biolegend) and Bcl2 (PeproTech, Rocky Hill, NJ,USA). The membranes were washed three times for 5 min with TBST and were then incubated with the appropriate secondary antibody in TBST with 5% nonfat milk for 1 h at room temperature. Protein binding was visualized using an enhanced chemiluminescence kit (Pierce) and X-ray films.

### Chromatin immunoprecipitation assays and real-time PCR

Chromatin immunoprecipitation assays were performed using a ChIP assay kit (Merck Millipore, Darmstadt, Germany) according to the manufacturer’s protocol. Cells (1 × 10^6^) were fixed in formaldehyde (1%) in medium and were then incubated for 10 min at room temperature. The pellets were washed with cold PBS, and the fixed cells were sonicated (15 cycles of 30-s sonication with 60 s of cooling in an ice-water bath) using a MISONIX XL-2000 (Qsonica, Newtown, CT, USA) at a power setting of 15, to shear the DNA in SDS lysis buffer. After preclearing by shaking with protein A or G, these lysates were incubated with 4 mg of antibodies or normal IgG at 4 °C for 16 h. The immuno-precipitated DNA was purified using a QIAquick PCR purification kit (Qiagen, Germany) according to the manufacturer’s protocol. Next, the purified DNA was used as a template for semi-quantitative and real-time PCRs with specific pairs of primers: for the mouse Aven promoter, 5′- TTT GAG CCA AGG TTC TAA CAA A -3′ and 5′ – CCA ATA CTA ACA TCA CGG AGG G -3′

### Detection of cell apoptosis by flow cytometry

Cell apoptosis was detected using the Annexin V-FITC/PI apoptosis detection kit. SH-SY5Y cells were seeded into 6-well culture plates (4.0 × 10^3^ cells/cm^2^) and cultured for 24 h. The medium was removed after these groups were treated, and the cells were rinsed once with 0.1 M PBS. Cells were passaged using 0.25% trypsinization for 2 min and collected in centrifuge tubes. Cells were centrifuged at 1500 r/min for 5 min, and the supernatants were discarded. Binding Buffer (200 μL) was added into each tube and vortexed. Annexin V-FITC (5 μL) was added for 10 min at room temperature in the dark, and the tubes were centrifuged at 1000 r/min for 5 min. Supernatants were discarded, and 200 μL Binding Buffer was added to resuspend the cells. PI (5 μL) was added to the mixture for testing.

### MTT assay

Cells were cultured in a 96-well culture plate at a density of 1 × 10^4^ cells/cm^2^ for 1–6 days. Each group was pretreated with different reagents for 4 h. Reagents and 40 μM TBHP were added, and 24 h later, the media were removed. DMEM (100 μL) with 10% MTT was added to each well and kept at 37 °C; 4 h later, the media was discarded. DMSO was added and placed on a shaker in the dark for 10 min. The absorption was measured at 570 nm using a Bio-Rad 400 microplate reader (Bio-Rad, Hercules, CA, USA). Experiments were repeated 3 times.

### Statistical analyses

All data were analyzed using the SPSS 17.0 statistical software (version 17.0, Chicago, IL, USA). The data are presented as the mean ± s.d. of three independent experiments; Student’s *t-test* was used for comparisons. *P <* 0.01 was considered significant.

## Results

### Foxo1 regulates CD127 expression in Treg cells

**Fig. 1:**
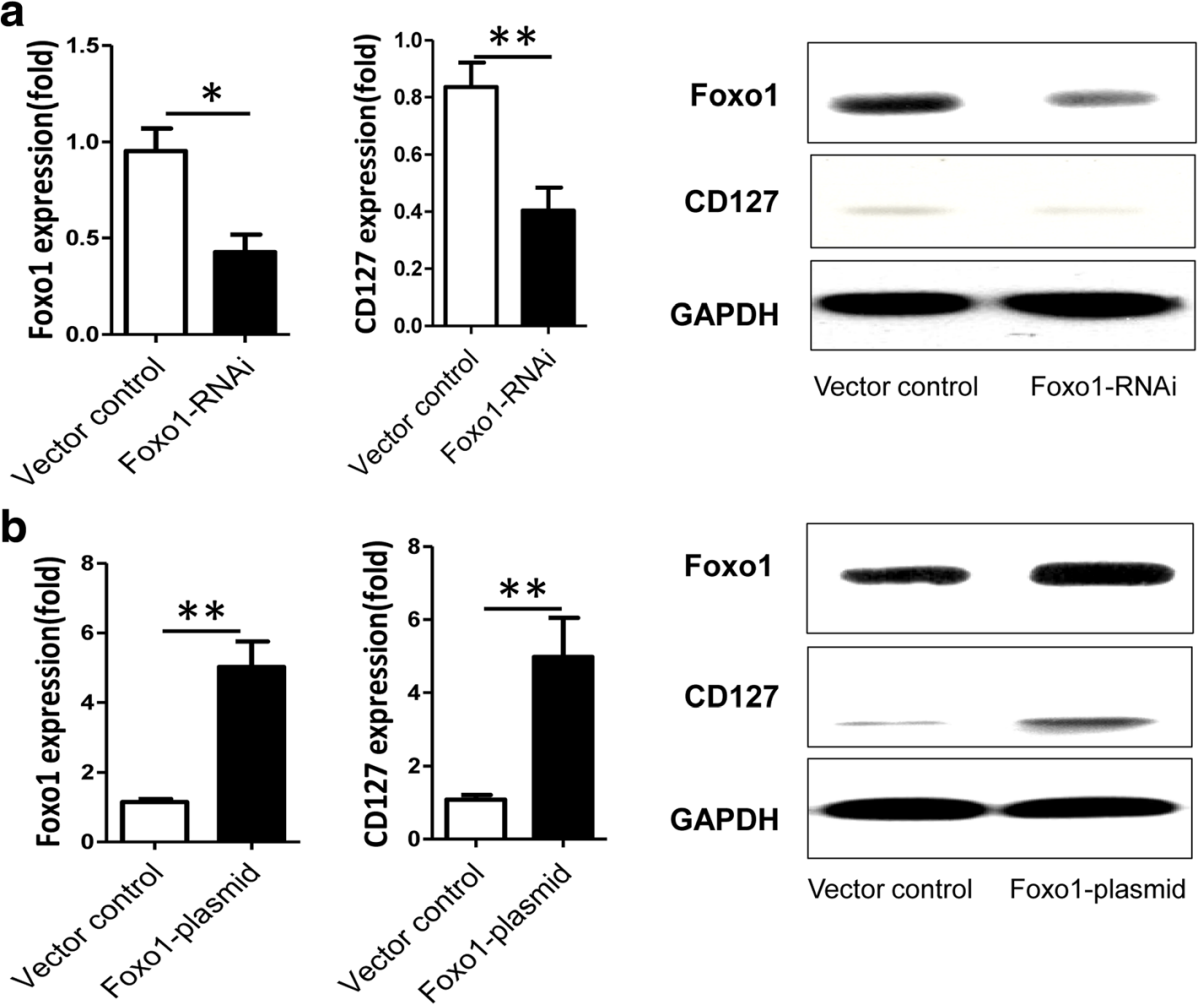
Detection of Foxo1 and CD127 after knockdown and over-expression of Foxo1 in Treg cells. **a** Expression of mRNA and protein of Foxo1 and CD127 in Treg cells, 48 h after transfection with Foxo1 siRNA. **b** Expression of mRNA and protein of Foxo1 and CD127 in Treg cells 48 h after transfection with over-expression plasmid of Foxo1. Treg cells stimulated with anti-CD3 (0.01 μg/ml) and anti-CD28 (1.0 μg/ml) in medium during culture. Data are presented as the mean + standard deviation (SD). *P < 0.01; **P < 0.005

**Fig. 2:**
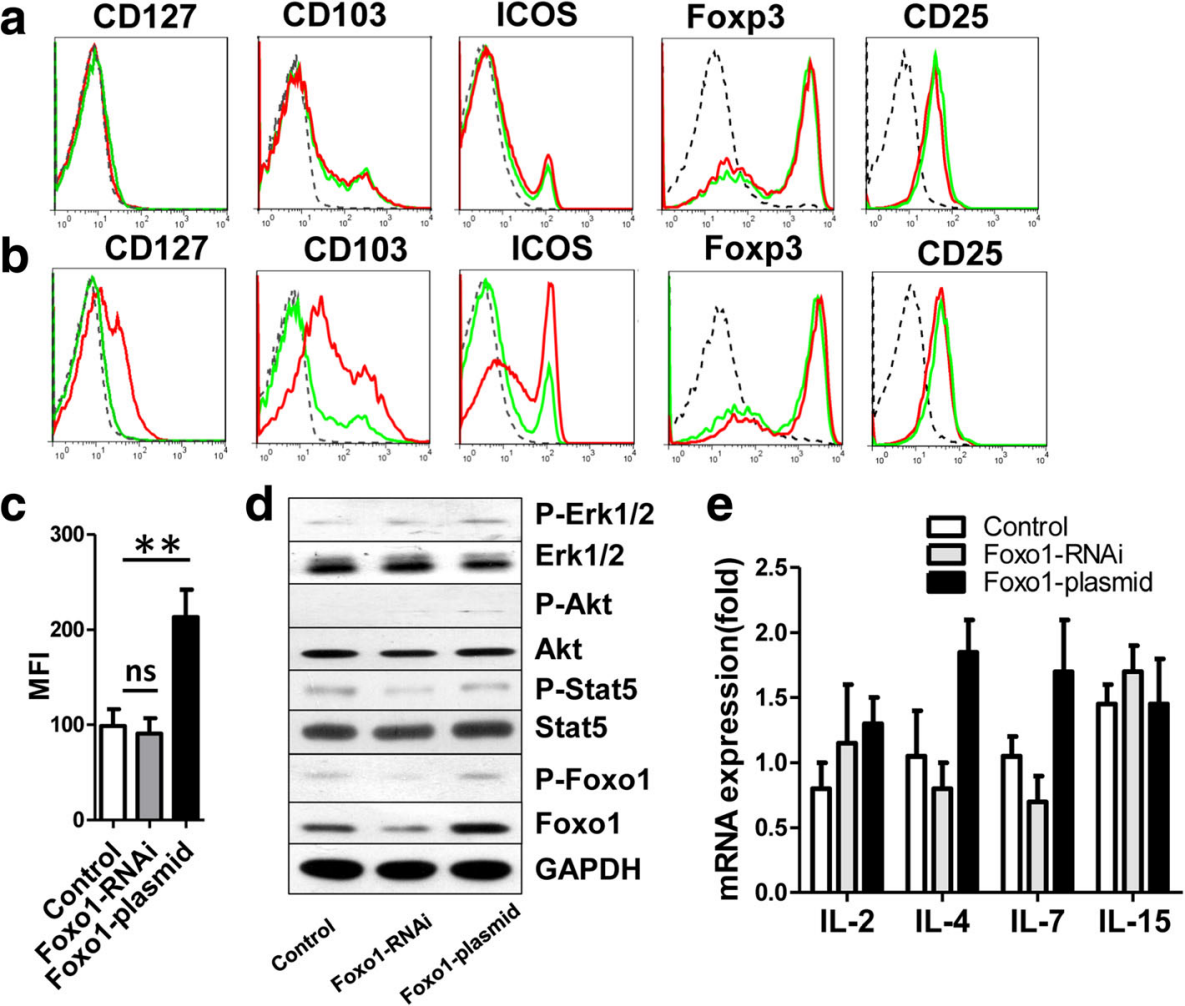
Detection of cell surface molecules and signaling pathway molecules after knockdown and over-expression of Foxo1 in Treg cells. **a**, Representative expression of the Foxo1, CD127, CD103, ICOS, Foxp3 or CD25 in Treg cells 48 h after transfection with Foxo1 siRNA by flow cytometry (broken black line: isotype, green line: control, red line: Foxo1 siRNA). **b** Representative expression of the Foxo1, CD127, CD103, ICOS, Foxp3 or CD25 in Treg cells 48 h after transfection with over-expression plasmid of Foxo1 by flow cytometry (broken black line: isotype, green line: control, red line: Foxo1 over-expression). **c** Detected of Median Fluorescence Intensity (MFI) for CD127 in Treg cells 48 h after transfection with Foxo1 siRNA and Foxo1 over-expression plasmid by flow cytometry. **d** Representative western blot of p-Erk1/2, total Erk1/2, p-Akt, total Akt, p-Stat5, total Stat5, p-Foxo1 and total Foxo1 in Treg cells 48 h after transfection with Foxo1 siRNA and over-expression plasmid of Foxo1, GADPH was used as a control. Treg cells stimulated with anti-CD3 (0.01 μg/ml) and anti-CD28 (1.0 μg/ml) in medium during culture. **e** Expression of mRNA for IL-2, IL-4, IL-7 and IL-15 in Treg cells, 48 h after transfection with Foxo1 siRNA and Foxo1 over-expression plasmid. All experiments were repeated at least three times. **P < 0.005, n.s: no significance

To investigate the role of Foxo1 on IL-7Rα expression in Treg cells, Treg cells were transfected with Foxo1 siRNA or control siRNA. We found that Foxo1 mRNA expression decreased by more than 50% when using Foxo1 siRNA compared to control siRNA (Fig. 1a). Consistent with these findings, Foxo1 protein expression was downregulated in Treg cells that were treated with Foxo1 siRNA compared with control siRNA (Fig. 1a). Unexpectedly, CD127 protein expression was downregulated in Treg cells-treated with Foxo1 siRNA compared with control siRNA (Fig. 1a). Conversely, Foxo1 over-expression increased the expression of CD127 in Treg cells (Figs. 1b and 2b), which suggested that Foxo1 plays an important role in CD127 expression.

### Foxo1 controls Treg cell proliferation by regulating CD127 expression

To test the role of Foxo1 in activating Treg cells, we detected CD103 and inducible co-stimulatory molecule (ICOS) by FCM, they have been described to identify activated Treg cells [22, 23]. CD127 was also detected by FCM in Treg cells. We found that CD127, CD103 and ICOS showed little change in Treg cells treated with Foxo1 siRNA and control siRNA, and the Median Fluorescence Intensity (MFI) of CD127 showed no significant difference between Foxo1 siRNA-treated cells and control siRNA cells (Fig. 2c). However, CD127, CD103 and ICOS expression was significantly increased in Foxo1 over-expression Treg cells, and the MFI of CD127 in Foxo1 over-expressed cells was 2.6 times higher than control (Fig. 2c). CD25 and Foxp3 showed little change in Treg cells in both Foxo1 knockdown and over-expressed cells (Fig. 2a, b). In addition, intracellular signaling molecules associated with the activity of Treg cells, including p-Erk1/2, p-Akt, p-Foxo1 and p-Stat5, demonstrated no change (Fig. 2d). These findings suggested that Treg cells can be activated by over-expression Foxo1. Cytokines that affect Treg cell activities, such as IL-2, IL-4, IL-7 and IL-15 [24], were detected using quantitative PCR. However, at the mRNA level of the genes, there were no differences when Foxo1 was over-expressed and knocked down (Fig. 2e).

**Fig. 3:**
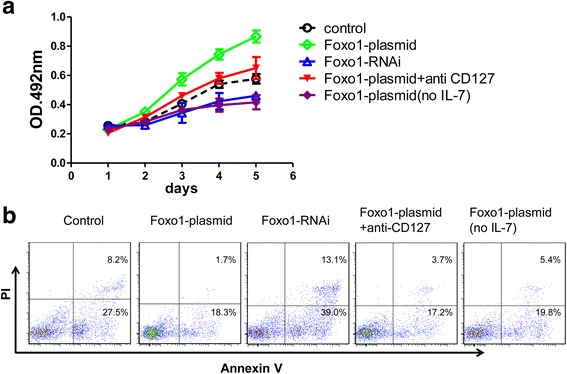
Proliferation and apoptosis after knockdown or over-expression of Foxo1 in Treg cells. **a** Treg cells growth were measured for 6 days using a MTT assay. Treg cells of every group (control, Foxo1-plasmid, Foxo1-RNAi and Foxo1-plasmid + CD127) except Foxo1-plasmid (no IL-7) was stimulated with anti-CD3 (0.01 μg/ml), anti-CD28 (1.0 μg/ml) and IL-7 (0.03 μg/ml) during culture, and anti-CD3 and anti-CD28 was only added in the Foxo1-plasmid (no IL-7) group. In addition, anti-CD127 (1:500) was added to the Foxo1-plasmid + CD127 group. These results are presented as the mean ± s.d. of the values obtained in three independent experiments. **b** Representative result of apoptosis in Treg cells based on the above groups 48 h after stimulation

Next, Treg cells were stimulated with CD3, CD28 and IL-7 to activate cell proliferation, and the cell proliferation rate was detected using ELISA. We found that the cell proliferation rate was higher in the Foxo1 over-expression cells than in the Foxo1 siRNA cells (Fig. 3a). But the cell proliferation rate decreased following treatment with the anti-CD127 antibody in the Foxo1 over-expression cells (Fig. 3a). Similarly, Treg cells were incubated without IL-7, the cell proliferation rate decreased (Fig. 3a), which suggested that Foxo1 controls Treg cell proliferation by regulating IL-7/CD127 signaling.

### Foxo1 controls Treg cell apoptosis by regulating Aven expression

**Fig. 4:**
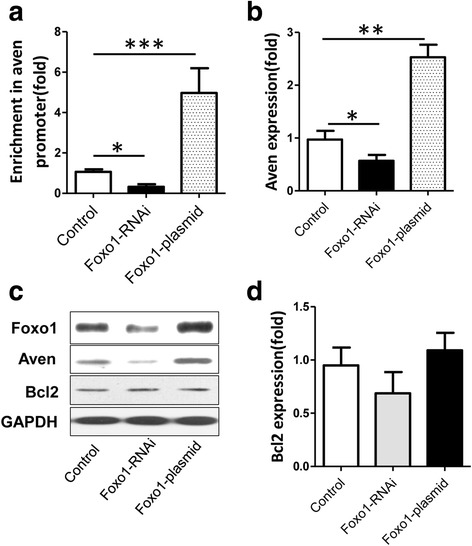
Detection of Aven after knockdown and over-expression of Foxo1 in Treg cells. **a** Level of Foxo1 enrichment at the Aven gene promoters in Treg cells in which Foxo1 was knocked down and over-expressed. Expression of the (**b**) mRNA and (**c**) protein of Foxo1 and Aven in Treg cells 48 h after transfection with Foxo1 siRNA and over-expression. Expression of the (**c**) protein and (**d**) mRNA of Bcl2 in Treg cells 48 h after transfection with Foxo1 siRNA and over-expression. Cells was stimulated with anti-CD3 (0.01 μg/ml), anti-CD28 (1.0 μg/ml) and IL-7 (0.03 μg/ml) during culture. Data are presented as the mean + standard deviation (SD). *P < 0.01; **P < 0.005, ***P < 0.001

Treg cells were transfected with Foxo1-plasmid prior to incubation with CD3, CD28 and IL-7, and apoptosis was measured according to the instructions of the PI-Annexin V apoptosis detection kit. We found that Foxo1 over-expression inhibited apoptosis (Fig. 3b). Conversely, cell apoptosis was enhanced in Treg cells after transfection with Foxo1 siRNA (Fig. 3b). However, IL-7 or CD127 demonstrated no apoptotic role in the presence of Foxo1 (Fig. 3b), suggesting that Foxo1 inhibits Treg cell apoptosis via other signaling pathways. In view of the opinion that Foxo1 may regulate the expression of the anti-apoptotic protein Aven [17], we tested the hypothesis that Foxo1 inhibits Treg cell apoptosis by regulating Aven expression. We first observed that Foxo1 can bind to the promoter of Aven as assessed by the Chip assay (Fig. 4a). Furthermore, we found that Aven mRNA expression was inhibited in Treg cells treated with Foxo1 siRNA compared with control (Fig. 4b). Conversely, Foxo1 over-expression increased the mRNA levels of Aven in Treg cells (Fig. 4b). Consistent with these findings, the protein expression of Aven showed the same trend at the mRNA level (Fig. 4c). This finding indicated that Foxo1 regulates Aven expression. The IL-7/CD127 axis is important for T-cell homeostasis, which can be also mediated by Bcl2. We detected the mRNA and protein levels of Bcl2 when Aven was over-expressed and knocked down, whereas at the mRNA and protein levels, there were no differences between the conditions (Fig. 4c,d). Bcl2 might be not regulated by Foxo1.

**Fig. 5:**
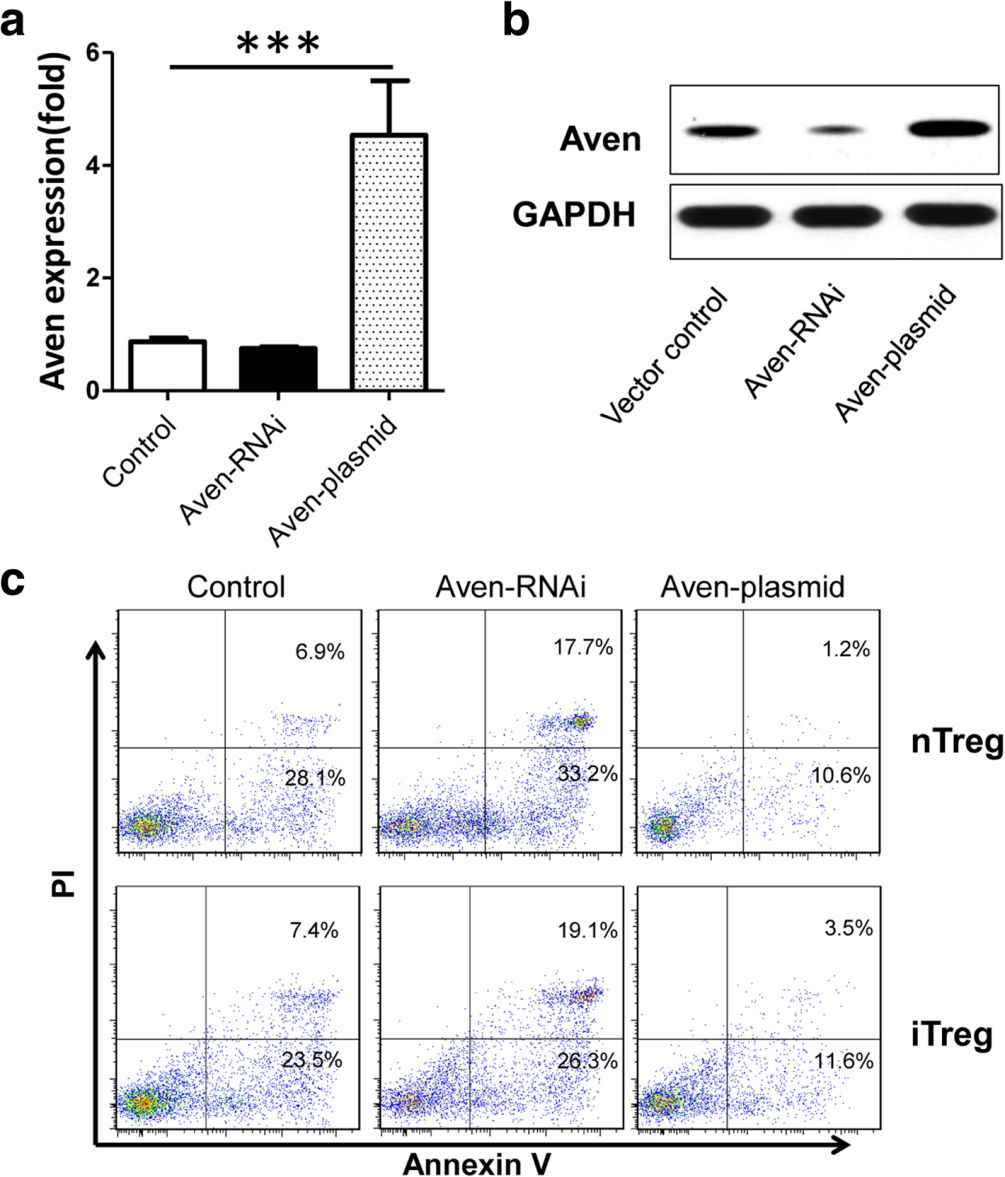
Detection of apoptosis after knockdown and over-expression of Aven in Treg cells. Expression of the (**a**) mRNA and (**b**)protein of Aven in Treg cells 48 h after transfection with Aven siRNA or over-expression plasmid of Foxo1. **c** Representative result of apoptosis in nTreg and iTreg cells 48 h after transfection with Aven siRNA or over-expression plasmid of Foxo1. Treg cells were stimulated with anti-CD3 (0.01 μg/ml) and anti-CD28 (1.0 μg/ml) in medium during culture. Data are presented as the mean + standard deviation (SD). ***P <0.001

To detect the role of Aven in Treg cell apoptosis, Treg cells were transfected with Aven siRNA or control siRNA. Compared to Treg cells that were treated with control siRNA, Aven expression was downregulated in Treg cells treated with Aven siRNA at the mRNA and protein levels (Fig. 5a-b). The flow cytometry results showed that apoptosis was enhanced in Treg cells transfected with Aven siRNA (Fig. 5c). When Aven was over-expressed in Treg cells, we found that Aven expression was upregulated at the mRNA and protein levels (Fig. 5a-b) and that cell apoptosis was attenuated (Fig. 5c), suggesting that Aven has anti-apoptotic properties in Treg cells. Furthermore, Treg cells are mainly divided into two subgroups: nTreg and iTreg. We also investigated the apoptosis of iTregs with Aven over-expression and knockdown. These results indicated that iTreg cells were similar with nTreg cells, such that apoptosis in both cell types could be regulated by Aven (Fig. 5c).

## Discussion

The forkhead box (Fox) family genes have important functions in many biological processes in human and mice. The mutations of Fox genes caused many human diseases, such as glaucoma, cancer, language disorders and immune diseases [25]. Foxn1, a member of the Fox family, involved in the differentiation and development of human central nervous system [26], and palys important roles for the differentiation and growth of T cell. Alterations of Foxn1 result in the failure of generating mature and functional thymocytes [27].

Foxo1 was also an important regulatory factor for the differentiation and function of T cells [28]. In the present study, we showed that Foxo1 activated the IL-7/CD127 signaling pathway, resulting in increased Treg proliferation. In addition, Foxo1 activated Aven expression, which plays an important role in cell apoptosis in Treg cells, suggesting that Foxo1 inhibits Treg cell apoptosis via Aven expression. These results indicated that Foxo1 plays a key role in controlling Treg cell proliferation and apoptosis by regulating CD127 and Aven expression.

Many studies have shown that Foxo1 plays an important role in T-cell homeostasis [19, 29]. Foxo1 regulates the expression of CD127 by binding to the promoter of the CD127 gene, and deletion of the Foxo1 gene can block the expression of CD127 in T-cells [19, 29]. In this study, we found that Foxo1 regulates CD127 expression in Treg cells. This result is consistent with the result obtained in previous reports. Activation of the IL-7/CD127 signaling pathway can promote Treg cell proliferation. Therefore, Foxo1 is involved in the regulation of Treg cell proliferation by regulating CD127.

In addition, we found that Foxo1 regulates cell apoptosis in Treg cells. However, CD127 was confirmed to not be involved in the cell apoptosis in Treg cells. Therefore, Foxo1 regulates other molecules to inhibit cell apoptosis. Aven is a novel molecule and can bind to the proapoptotic APAF-1 protein to prevent the oligomerization of APAF-1 in the intrinsic apoptosis pathway [30]. Recently, It was shown that Aven contributes to the anti-apoptotic properties of this protein [20]. We found that Foxo1 can regulate Aven expression in Treg cells, which inhibits cell apoptosis. These data indicated that Foxo1 is involved in the regulation of Treg cell apoptosis via Aven.

In conclusion, we demonstrated that Foxo1 over-expression in the Treg cells results in a significant enhancement of cell proliferation due to the activation of the IL-7/CD127 signaling pathway. In addition, Foxo1 over-expression in Treg cells inhibits cell apoptosis by regulating Aven expression. Collectively, Foxo1 is required for Treg cell proliferation and apoptosis via CD127 and Aven expression.

## Conclusions

Our results suggest that Foxo1 is a positive regulatory factor for nTreg cell activation. It promotes the proliferation of Treg cells and inhibits apoptosis via the Aven signaling pathway. These data suggest that Foxo1 might be a promising target for activating nTreg cells in vivo and in vitro. Basic and clinical research of treatment drugs provides a useful reference.

## Abbreviations

Foxo1: Forkhead box protein o 1
GVHD: Graft-versus-host disease
IL-7: Interleukin-7
IL-7R: Interleukin-7 receptor
Treg: Regulatory T

## References

[1] Curiel TJ (2007). Tregs and rethinking cancer immunotherapy. J Clin Invest.

[2] Ono M, Yaguchi H, Ohkura N, Kitabayashi I, Nagamura Y, Nomura T, Miyachi Y, Tsukada T, Sakaguchi S (2007). Foxp3 controls regulatory T-cell function by interacting with AML1/Runx1. Nature.

[3] Ersvaer E, Liseth K, Skavland J, Gjertsen BT, Bruserud O (2010). Intensive chemotherapy for acute myeloid leukemia differentially affects circulating TC1, TH1, TH17 and TREG cells. BMC Immunol.

[4] Chen W (2011). Tregs in immunotherapy: opportunities and challenges. Immunotherapy.

[5] La Cava A (2009). Natural Tregs and autoimmunity. Front Biosci.

[6] Oleinika K, Nibbs RJ, Graham GJ, Fraser AR (2013). Suppression, subversion and escape: the role of regulatory T cells in cancer progression. Clin Exp Immunol.

[7] Schliesser U, Streitz M, Sawitzki B (2012). Tregs: application for solid-organ transplantation. Curr Opin Organ Transplant.

[8] Sakaguchi S, Fukuma K, Kuribayashi K, Masuda T (1985). Organ-specific autoimmune diseases induced in mice by elimination of T cell subset. I. Evidence for the active participation of T cells in natural self-tolerance; deficit of a T cell subset as a possible cause of autoimmune disease. J Exp Med.

[9] Waldmann H, Adams E, Fairchild P, Cobbold S (2006). Infectious tolerance and the long-term acceptance of transplanted tissue. Immunol Rev.

[10] Gorczynski RM (2006). Thymocyte/splenocyte-derived CD4 + CD25 + Treg stimulated by anti-CD200R2 derived dendritic cells suppress mixed leukocyte cultures and skin graft rejection. Transplantation.

[11] Edinger M, Hoffmann P, Ermann J, Drago K, Fathman CG, Strober S, Negrin RS (2003). CD4 + CD25+ regulatory T cells preserve graft-versus-tumor activity while inhibiting graft-versus-host disease after bone marrow transplantation. Nat Med.

[12] Atanackovic D, Cao Y, Luetkens T, Panse J, Faltz C, Arfsten J, Bartels K, Wolschke C, Eiermann T, Zander AR (2008). CD4 + CD25 + FOXP3+ T regulatory cells reconstitute and accumulate in the bone marrow of patients with multiple myeloma following allogeneic stem cell transplantation. Haematologica.

[13] MacDonald KG, Hoeppli RE, Huang Q, Gillies J, Luciani DS, Orban PC, Broady R, Levings MK (2016). Alloantigen-specific regulatory T cells generated with a chimeric antigen receptor. J Clin Invest.

[14] Peffault de Latour R, Dujardin HC, Mishellany F, Burlen-Defranoux O, Zuber J, Marques R, Di Santo J, Cumano A, Vieira P, Bandeira A (2006). Ontogeny, function, and peripheral homeostasis of regulatory T cells in the absence of interleukin-7. Blood.

[15] Mazzucchelli R, Hixon JA, Spolski R, Chen X, Li WQ, Hall VL, Willette-Brown J, Hurwitz AA, Leonard WJ, Durum SK (2008). Development of regulatory T cells requires IL-7Ralpha stimulation by IL-7 or TSLP. Blood.

[16] Carrette F, Surh CD (2012). IL-7 signaling and CD127 receptor regulation in the control of T cell homeostasis. Semin Immunol.

[17] Ouyang W, Liao W, Luo CT, Yin N, Huse M, Kim MV, Peng M, Chan P, Ma Q, Mo Y (2012). Novel Foxo1-dependent transcriptional programs control T(reg) cell function. Nature.

[18] Hedrick SM, Hess Michelini R, Doedens AL, Goldrath AW, Stone EL (2012). FOXO transcription factors throughout T cell biology. Nat Rev Immunol.

[19] Ouyang W, Beckett O, Flavell RA, Li MO (2009). An essential role of the Forkhead-box transcription factor Foxo1 in control of T cell homeostasis and tolerance. Immunity.

[20] Melzer IM, Fernandez SB, Bosser S, Lohrig K, Lewandrowski U, Wolters D, Kehrloesser S, Brezniceanu ML, Theos AC, Irusta PM (2012). The Apaf-1-binding protein Aven is cleaved by Cathepsin D to unleash its anti-apoptotic potential. Cell Death Differ.

[21] Nakae J, Kitamura T, Silver DL, Accili D (2001). The forkhead transcription factor Foxo1 (Fkhr) confers insulin sensitivity onto glucose-6-phosphatase expression. J Clin Invest.

[22] Lund JM, Hsing L, Pham TT, Rudensky AY (2008). Coordination of early protective immunity to viral infection by regulatory T cells. Science.

[23] Scott-Browne JP, Shafiani S, Tucker-Heard G, Ishida-Tsubota K, Fontenot JD, Rudensky AY, Bevan MJ, Urdahl KB (2007). Expansion and function of Foxp3-expressing T regulatory cells during tuberculosis. J Exp Med.

[24] Yates J, Rovis F, Mitchell P, Afzali B, Tsang J, Garin M, Lechler RI, Lombardi G, Garden OA (2007). The maintenance of human CD4+ CD25+ regulatory T cell function: IL-2, IL-4, IL-7 and IL-15 preserve optimal suppressive potency in vitro. Int Immunol.

[25] Hannenhalli S, Kaestner KH (2009). The evolution of Fox genes and their role in development and disease. Nat Rev Genet.

[26] Amorosi S, D’Armiento M, Calcagno G, Russo I, Adriani M, Christiano AM, Weiner L, Brissette JL, Pignata C (2008). FOXN1 homozygous mutation associated with anencephaly and severe neural tube defect in human athymic Nude/SCID fetus. Clin Genet.

[27] Pignata C, Fusco A, Amorosi S (2009). Human clinical phenotype associated with FOXN1 mutations. Adv Exp Med Biol.

[28] Carrette F, Fabre S, Bismuth G (2009). FOXO1, T-cell trafficking and immune responses. Adv Exp Med Biol.

[29] Kerdiles YM, Beisner DR, Tinoco R, Dejean AS, Castrillon DH, DePinho RA, Hedrick SM (2009). Foxo1 links homing and survival of naive T cells by regulating L-selectin, CCR7 and interleukin 7 receptor. Nat Immunol.

[30] Chau BN, Cheng EH, Kerr DA, Hardwick JM (2000). Aven, a novel inhibitor of caspase activation, binds Bcl-xL and Apaf-1. Mol Cell.

